# Microsurgical Clipping of a Posterior Inferior Cerebellar Artery Aneurysm Following Failed Pipeline Stent

**DOI:** 10.7759/cureus.13568

**Published:** 2021-02-26

**Authors:** Rishi Suresh, Amanda V Jenson, Gavin Britz

**Affiliations:** 1 Neurological Surgery, Texas A&M College of Medicine, Bryan, USA; 2 Neurological Surgery, Houston Methodist Hospital, Houston, USA; 3 Neurosurgery, Houston Methodist Neurological Institute, Houston, USA

**Keywords:** microsurgical aneurysm clipping, unruptured aneurysm

## Abstract

Aneurysms of the posterior inferior cerebellar artery (PICA) are rare, with limited consensus on appropriate management. These aneurysms have been noted to have a faster growth rate and are more prone to rupture. Accessing these aneurysms for microsurgical clipping is challenging, and has traditionally required significant removal of the occipital condyle, putting the patient at risk for future complications. Therefore, some have opted to utilize minimally invasive techniques such as a pipeline stent, though these methods can fail to cause complete occlusion of the aneurysm. The current case describes a patient who was found to have a PICA aneurysm that was initially managed with a pipeline stent. However, upon further follow up, the aneurysm showed continued filling, leading to the decision to clip the aneurysm. In this case, we describe the use of a far lateral approach for accessing and clipping a PICA aneurysm with minimal removal of the occipital condyle. The patient successfully tolerated the surgery and was discharged home.

## Introduction

Aneurysms of the posterior inferior cerebellar artery (PICA) are relatively rare, and there is limited information regarding appropriate management strategies of these lesions [1]. Relative to aneurysms of the anterior circulation, posterior circulation aneurysms have been described to grow faster and have higher rates of rupture [2]. Historically, aneurysms of the PICA were treated using microsurgical clipping. To access the aneurysm, the occipital condyle often had to be removed, causing many patients to experience craniocervical instability. In recent years, pipeline embolization has been shown to be a minimally invasive method of occluding the aneurysm [3-4]. The use of the pipeline stent has been shown to have reduced morbidity compared to microsurgical clipping [5]. However, in some instances, these devices can fail to completely occlude the aneurysm, leading to continued filling.

In the current case, we describe a patient with a PICA aneurysm that was initially managed with a pipeline stent. However, the aneurysm was noted to have continued filling on subsequent follow up digital subtraction angiogram (DSA), leading to the decision to clip the aneurysm. We describe the successful use of a far lateral approach for accessing the aneurysm with minimal removal of the occipital condyle.

## Case presentation

**Figure 1:**
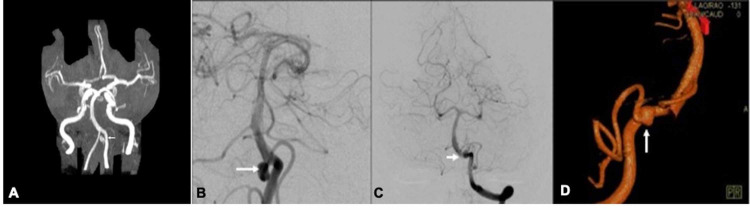
MRA and DSA of left PICA aneurysm. A. Coronal MRA showing left PICA aneurysm. B. Oblique DSA showing left PICA aneurysm. C. AP DSA showing left PICA aneurysm at origin of PICA. D. 3-D DSA showing irregular left PICA aneurysm measuring 5.7 mm x 4.6 mm x 5.15 mm. MRA, magnetic resonance angiogram; DSA, digital subtraction angiogram; PICA, posterior inferior cerebellar artery; AP, anteroposterior

A 72-year-old female with a past medical history of migraines and benign paroxysmal positional vertigo (BPPV) presented to the ED with the complaint of new onset numbness and tingling of her right face that eventually progressed to the left side. She also developed significant nausea which resolved with meclizine. A noncontrast CT scan of the brain was performed in the ED to rule out stroke and found no acute abnormalities. The patient’s neurologist was consulted and suggested that her symptoms might be caused by complex migraines and she was discharged. The patient then noticed that her right eye was beginning to have diminished vision over the week after she was discharged from the ED. She went to see her primary care physician who ordered an MRI study with and without contrast of the brain and a magnetic resonance angiogram (MRA). These images revealed an aneurysm at the left PICA origin (Figure 1A).

She was then referred for a digital subtraction angiogram (DSA) of the brain, commonly referred to as a cerebral angiogram. The cerebral angiogram was scheduled and took place a month later. The cerebral angiogram showed clearly an irregular aneurysm at the origin of the left PICA, 5.7 mm in size, with a wide neck (greater than 3.75 mm) (Figure 1B-D). The neurointerventionist who performed the diagnostic cerebral angiogram then scheduled her for treatment. A neurvas pipeline flex stent 4 mm x 14 mm was placed in the PICA to divert flow from the left PICA aneurysm. The patient took aspirin (81 mg) and clopidogrel (75 mg) prior to and following the procedure. In the evening following the stent placement, the patient experienced mild right homonymous hemianopsia that improved throughout the hospital course. She was discharged with complaints of seeing dark shadows in her right visual field in both eyes on post procedure day two and was referred for follow up with ophthalmology. Upon follow up with ophthalmology, she was noted to have resolving nonspecific visual field changes likely caused by multifocal occlusions during the stenting.

**Figure 2:**
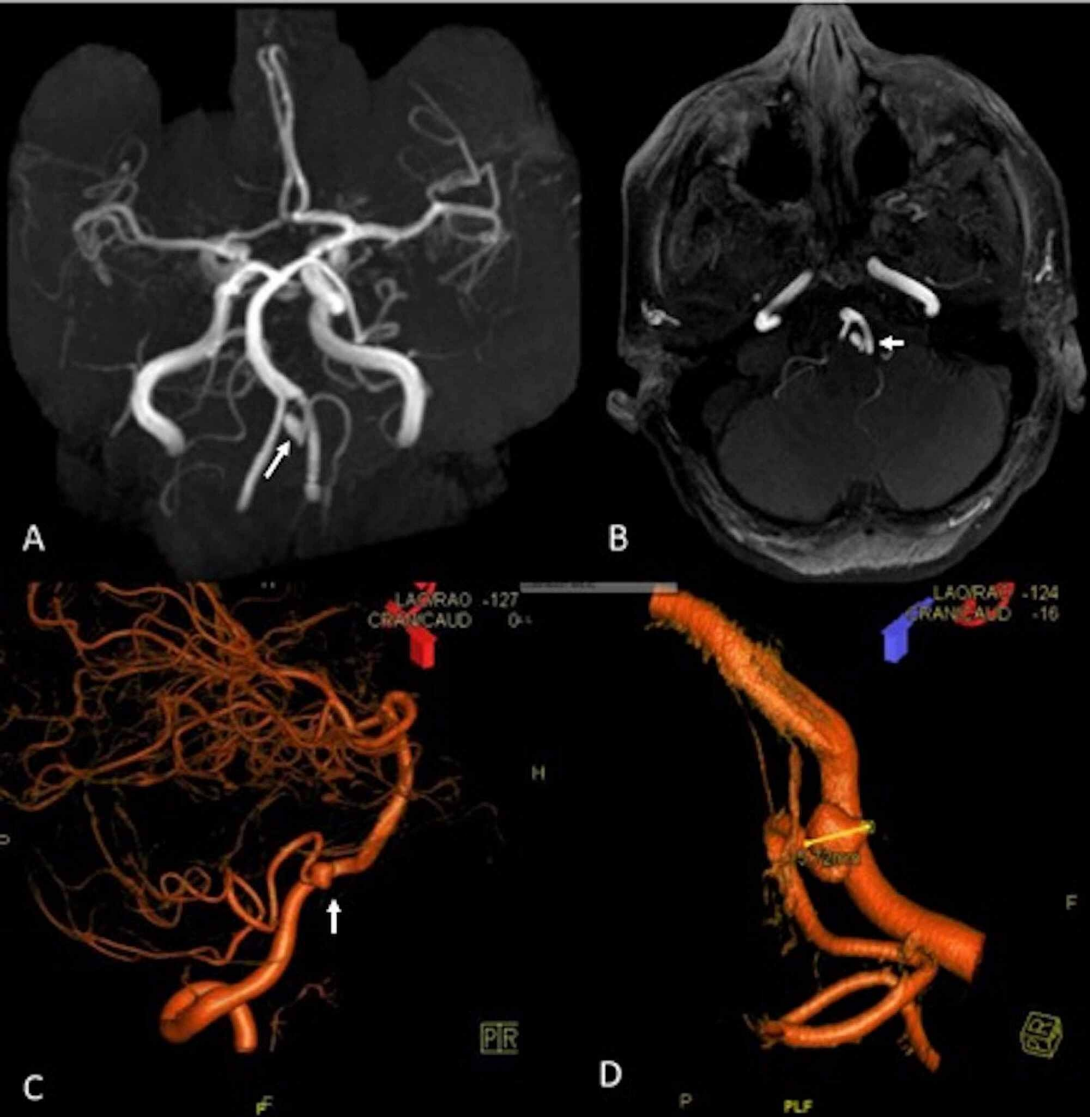
MRA of PICA aneurysm. A. Two-year follow up MRA shows residual filling of left PICA aneurysm. B. Axial MRA from two-year follow up showing residual filling of left PICA aneurysm. C. 3-D DSA lateral view of left vertebral artery run, showing residual filling of left PICA aneurysm. D. Close up of 3-D DSA showing increase of size at 5.72 mm. MRA, magnetic resonance angiogram; PICA, posterior inferior cerebellar artery; DSA, digital subtraction angiogram

A follow up cerebral angiogram was performed one year later. This showed some residual filling of the aneurysm with a slightly smaller size compared to the year prior (5.7 mm in greatest dimension with very narrow neck). At this time, the decision was made to observe the aneurysm. Additionally, her clopidogrel was discontinued, and she was counseled to continue taking aspirin daily (81 mg). An MRA was then done two years following the embolization. This imaging found the aneurysm to be slightly increased in size (now 6 mm) with continued filling, and the patient was recommended to obtain a cerebral angiogram (Figure 2). The cerebral angiogram showed continued filling of the aneurysm (Figure 2). Due to the persistence of flow into the aneurysm the decision was made to surgically clip the aneurysm.

**Figure 3:**
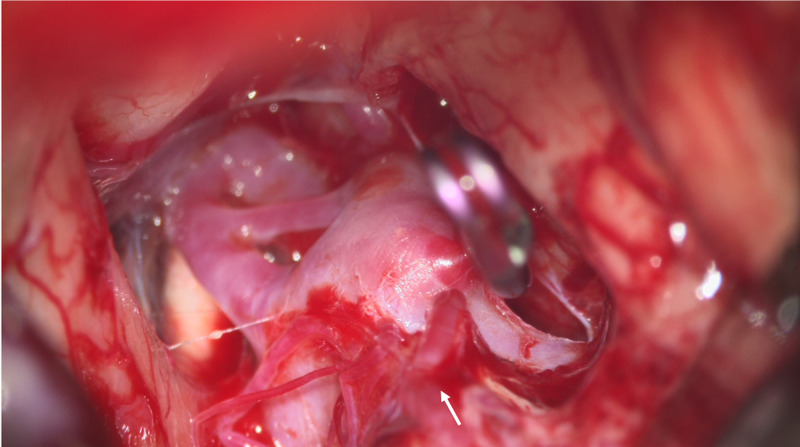
Intraoperative view of clip on left PICA aneurysm via far lateral approach.

The patient was taken for surgery later that year. Prior to positioning, a sheath was inserted in the left femoral artery for access during the case for an intraoperative angiogram. She was positioned in a three-quarter prone position with the left side up. The incision was marked out on midline feeling for the inion and C2 spinous process as anatomical landmarks, then carrying on in a curvilinear hockey-shaped fashion to end at the left mastoid. Local anesthetic was administered to the incision after cleansing with alcohol. The operative site was then prepped and draped in normal sterile manner with chlorhexidine. Sterile drapes were applied to the field. Incision was made with a number 10 blade to the subcutaneous fat. Monopolar electrocautery was then used to further dissect on the midline raphe from approximately C3 spinous process up to the inion. Further dissection was carried out to expose the left occipital skull base, foramen magnum, and C1 arch. An M8 drill was then used to perform a suboccipital craniotomy. The spinous processes of C1 and part of C2 were removed exposing the left extradural vertebral artery. Very little occipital condyle (approximately posterior one-third) was removed in this process. With the use of the microscope, the vertebral artery was then followed up to the origin of the left PICA. Using a telovelar approach, the cerebral tonsil was dissected and retracted medially. The PICA was then identified, and the pipeline stent could be visualized bridging the aneurysm. Adenosine (6 mg) cardiac arrest was then used to collapse the aneurysm and allow for better visualization, an approach that is commonly used by the lead surgeon when temporary vertebral clipping is not possible (due to inadequate surgical exposure). The aneurysm clip (11 mm x 3.5 mm x 6 mm) was then applied to the aneurysm, intraoperative doppler showed appropriate flow in the parent vessels (Figure 3). An intraoperative angiogram was then performed that showed the aneurysm was appropriately occluded. The incision was then closed in multiple layers with a running nylon on the skin. The patient was turned supine and the sheath was removed, pressure was held for 30 minutes. The patient awoke from surgery, tolerating the procedure well with no postoperative complications and was subsequently discharged on postoperative day seven.

## Discussion

The anatomy of the PICA is complex and often variable. Generally, the PICA comes off the vertebral artery and is divided into five distinct anatomical segments -- the anterior medullary (p1), lateral medullary (p2), tonsillomedullary (p3), telovelotonsillar (p4), and cortical (p5) [6]. The anterior medullary segment of the PICA runs anterior to the medulla, and then turns posteriorly through or near the hypoglossal rootlets to the border of the anterior and lateral surfaces of the medulla, consisting of the most prominent portion of the inferior olive. The lateral medullary segment of the PICA begins at the most prominent portion of the olive and terminates at the origin of the glossopharyngeal nerve [cranial nerve (CN) IX], vagus nerve (CN X), and spinal accessory nerve (CN XI) rootlets. The tonsillomedullary segment begins at the rootlets of CN IX, X, and XI and extends medially along the medulla to the caudal aspect of the tonsil, then ends at the midpoint of the medial surface of the tonsil. This segment often forms a loop in its course known as the infratonsillar loop. This characteristic loop of PICA is the easiest way to locate this artery on DSA. The telovelotonsillar segment originates about halfway through the ascent of the PICA along the medial tonsillar surface and extends into the roof of the fourth ventricle and terminates when it passes through the fissure near the tonsils and vermis. The cortical segment is the final segment of the PICA and originates at the groove between the tonsil and the vermis.

 Lesions of the PICA can cause a series of neurological deficits. The most well known being Wallenberg’s syndrome, a condition that leads to several characteristic clinical findings. Several structures are injured with a lesion of the PICA, including the spinothalamic tract, spinal trigeminal nucleus, nucleus ambiguous, inferior vestibular nucleus, and inferior cerebellar peduncle. Therefore, patients may have ipsilateral limb ataxia, vertigo, nausea/vomiting, dysarthria, dysphagia, ipsilateral loss of pain and temperature sensation from the face, contralateral loss of pain and temperature from the body, and ipsilateral features of Horner’s syndrome [7].

 Aneurysms of the posterior circulation are relatively rare, as approximately 80%-85% of intracranial aneurysms are located in the anterior circulation [8]. Posterior circulation aneurysms often involve the basilar artery bifurcation, or the junction of the vertebral artery and the PICA. Aneurysms of the PICA are encountered even less frequently, and account for approximately 0.5%-3% of all aneurysms [9]. While infrequent, posterior circulation aneurysms account for a significantly increased risk of rupture. In a systematic review evaluating the risk of rupture of intracranial aneurysms, Rinkel et al. identified that aneurysms of the posterior circulation have a relative risk of rupture of 4.1 compared to anterior cerebral artery (ACA) aneurysms [2]. The reason for this difference in rupture is not clear, but some have noted that altered hemodynamics in the posterior circulation with respect to wall shear stress might contribute to aneurysm growth and rupture [10]. Posterior circulation aneurysms have also been noted to have a higher growth rate at 3.8% per year for posterior circulation aneurysms, compared to 2.7% growth for anterior circulation aneurysms [11].

In an analysis of 83 PICA aneurysms, Rodriguez-Hernandez et al. found that the vast majority of these aneurysms (65.1%) arose proximally, while the remainder were located distally [1]. When considering distal PICA aneurysms specifically, they found that the majority originate from the lateral medullary (p2) or the tonsillomedullary segments (p3) [6]. Other studies have suggested that the major location for a PICA aneurysm is at the PICA-vertebral junction [12-13]. Hudgins et al. identified 17 to be originating from the PICA-vertebral artery junction [12]. In another study, Horowitz found that 79% of PICA aneurysms were located at the PICA origin [13].

The patient in this case initially underwent a pipeline embolization to manage her PICA aneurysm, a strategy which has shown promising results in the past few years [3-4]. Many times, management of PICA aneurysms using pipeline embolization devices (PED) involves placing the device in the vertebral artery. Recently, placement of the PED in the PICA itself has also been shown to be successful [5]. However, some have also shown in the literature that surgery can be an effective first-line management option for these aneurysms as well [9, 14-15]. Furthermore, as shown in this case, sometimes PEDs are not effective in completely occluding an aneurysm. In these situations, our case suggests that a far lateral approach can be used to clip the aneurysm as a potential second line treatment.

 In the current case, very little occipital condyle was removed in the access of the aneurysm. Removal of the occipital condyle has been traditionally performed to increase visibility. In an analysis of far-lateral approaches to the foramen magnum for meningioma resection, Nanda et al. described that while partial removal of the occipital condyle was not critical for complete resection, removal of one third of the occipital condyle was associated with an increase of 15.9 degrees of visibility [16]. However, while providing increased visibility in the surgical field, removal of the occipital condyle may place the patient at increased risk for craniocervical junction instability depending on the amount of condyle removed [17]. Indeed, excess removal of the occipital condyle has been shown to increase the range of motion between C0 and C2, and a fusion may become necessary to provide stabilization [18]. Additionally, Di Somma et al. describe a series of vertebral artery-PICA aneurysms that were managed successfully with a far-lateral approach in which the occipital condyle was left intact, further suggesting that these aneurysms can be managed with minimal condyle disruption [19]. Furthermore, excess removal of the occipital condyle can place the patient at risk for hypoglossal nerve or vertebral artery damage, and can lead to significant postoperative pain. Specifically in the management of PICA aneurysms, Seoane et al. found that drilling of the occipital condyle was not necessary for access, and may increase the risk of bleeding and prolong surgical time [20].

## Conclusions

Aneurysms of the PICA are fairly rare, and can be challenging to manage. Common approaches include the use of a pipeline flow-diverting stent or microsurgical clipping. Pipeline stents are often attractive options due to their minimally invasive nature. However, as shown in the current case, these devices can potentially fail to completely occlude the aneurysm. In such cases, surgical management is required. In this case, we describe the use of a far lateral approach for management of the PICA aneurysm. Far lateral approaches for PICA clipping often involve removal of the occipital condyle, which can place the patient at risk for craniocervical junction instability postoperatively. However, in the current case, we describe that the far lateral approach can be accomplished with little to no bony removal of the occipital condyle as to not disturb the articulation with the superior facet of the C1 vertebral arch. Such an approach allows for successful aneurysm clipping while minimizing postoperative complications.
